# Comprehensive Molecular Serology of Human *Chlamydia trachomatis* Infections by Peptide Enzyme-Linked Immunosorbent Assays

**DOI:** 10.1128/mSphere.00253-18

**Published:** 2018-08-01

**Authors:** K. Shamsur Rahman, Toni Darville, Ali N. Russell, Catherine M. O'Connell, Harold C. Wiesenfeld, Sharon L. Hillier, De’Ashia E. Lee, Bernhard Kaltenboeck

**Affiliations:** a Department of Pathobiology, College of Veterinary Medicine, Auburn University, Auburn, Alabama, USA; b Department of Pediatrics, University of North Carolina at Chapel Hill, Chapel Hill, North Carolina, USA; c Department of Obstetrics, Gynaecology and Reproductive Sciences, the University of Pittsburgh School of Medicine and the Magee-Womens Research Institute Pittsburgh, Pennsylvania, USA; Parasitology Services

**Keywords:** B cell epitopes, *Chlamydia pneumoniae*, *Chlamydia trachomatis*, IgG1, IgG3, IgA1, antibody detection, antibody isotype, cross-reactivity, diagnosis, multipeptide ELISA, peptide antigens, serology, species specific

## Abstract

Sensitive species-specific detection of anti-Chlamydia trachomatis antibodies is compromised by cross-reactivity of the C. trachomatis antigens used in standard microimmunofluorescence (MIF) testing and enzyme-linked immunosorbent assays (ELISAs). Previously, we discovered 48 strongly reactive C. trachomatis-specific B cell epitope peptides from 21 immunodominant proteins. Here we comprehensively evaluated the 11 top-ranked C. trachomatis-specific peptide antigens from 8 proteins for use in *C. trachomatis* serology. Sera from 125 women with nucleic acid amplification test (NAAT)-confirmed active C. trachomatis infection and from 49 healthy women with a low risk of C. trachomatis infection were used as anti-C. trachomatis antibody-positive and -negative sera. Results obtained for detection of IgG1, IgG3, and IgA1 antibodies against the 11 C. trachomatis peptide antigens were compared to results from 4 commercial anti-C. trachomatis IgG ELISAs. Using composite reference standards (CRS) of all assays for anti-C. trachomatis antibody status, commercial ELISAs detected antibodies in antibody-positive women with sensitivities of 51.5% to 64.8%. In contrast, a combination of the results of all 11 peptides detected IgG (IgG1 and IgG3) antibodies with 91.8% sensitivity, and a labor-saving combination of the 5 optimal peptides still detected antibodies in antibody-positive women with 86.5% sensitivity (all at 98% specificity). The superior performance of the combined peptide ELISAs was confirmed by area under the receiver operating characteristic curve (ROC-AUC), likelihood ratio, and predictive value analyses. The higher sensitivity of the peptide assays results from using multiple B cell epitopes of several C. trachomatis immunodominant proteins, including OmpA, compared to exclusively using the OmpA antigens used in commercial ELISAs. Thus, ELISAs with combined use of synthetic peptide antigens for C. trachomatis antibody detection have the advantage of simultaneous high sensitivity and high specificity.

**IMPORTANCE** For detection of anti-Chlamydia trachomatis antibodies by serological assays, use of classical whole-organism chlamydial antigens results in high cross-reactivity. These antigens bind mainly antibodies against the major outer membrane protein (OmpA) and bind antibodies against other immunodominant non-OmpA proteins to a lesser extent, resulting in poor assay sensitivity. The specificity of *C. trachomatis* serology is also compromised by the high prevalence of cross-reactive anti-C. pneumoniae antibodies in human populations. We previously identified 48 highly specific C. trachomatis B cell epitope peptide antigens of 21 immunodominant proteins. This study validated peptide antigen-based novel ELISAs that provide highly specific and sensitive detection of anti-C. trachomatis antibodies. Compared to four commercial ELISAs that achieved only poor sensitivities (51.5% to 64.8%), the combined signals of 5 to 11 peptides provided high sensitivity (86.5% to 91.8%) at the same 98% specificity. Thus, by using multiple peptide antigens of immunodominant proteins, we created simple ELISAs with specificity and sensitivity superior to standard *C. trachomatis* serodiagnosis.

## Abstract (precis)

For detection of anti-Chlamydia trachomatis antibodies by serological assays, use of classical whole-organism chlamydial antigens results in high cross-reactivity. These antigens bind mainly antibodies against the major outer membrane protein (OmpA) and bind antibodies against other immunodominant non-OmpA proteins to a lesser extent, resulting in poor assay sensitivity. The specificity of *C. trachomatis* serology is also compromised by the high prevalence of cross-reactive anti-C. pneumoniae antibodies in human populations. We previously identified 48 highly specific C. trachomatis B cell epitope peptide antigens of 21 immunodominant proteins. This study validated peptide antigen-based novel ELISAs that provide highly specific and sensitive detection of anti-C. trachomatis antibodies. Compared to four commercial ELISAs that achieved only poor sensitivities (51.5% to 64.8%), the combined signals of 5 to 11 peptides provided high sensitivity (86.5% to 91.8%) at the same 98% specificity. Thus, by using multiple peptide antigens of immunodominant proteins, we created simple ELISAs with specificity and sensitivity superior to standard *C. trachomatis* serodiagnosis.

## INTRODUCTION

Of 11 *Chlamydia* spp., C. trachomatis and C. pneumoniae are mainly pathogens for human hosts (1, 2). *C. trachomatis* serovars A to C cause ocular infection, serovars D to K cause genitourinary tract infections, and serovars L1 to L3 cause lymphogranuloma venereum (1, 3). In contrast, the single human serovar of C. pneumoniae causes respiratory disease infections such as pharyngitis, bronchitis, and community-acquired pneumonia (2). For understanding and diagnosing C. trachomatis infections and diseases, highly specific serological assays are urgently needed for complementation of pathogen detection assays (4). Anti-C. trachomatis antibodies have the power to indicate recent exposure as well as a history of exposure to C. trachomatis (4–7). However, cross-reactivity caused by antibodies elicited during highly prevalent C. pneumoniae respiratory infections in children (8, 9, 76) complicates results of serological studies for C. trachomatis, emphasizing the need for C. trachomatis species-specific serological assays.

The microimmunofluorescence (MIF) test for detection of C. trachomatis-specific antibodies has remained the gold standard since its introduction (4, 10–12). MIF is performed as an indirect fluorescent antibody technique that enables microscopic observation of captured antibody on fixed chlamydial elementary body (EB) antigens (10–12). Due to the presence of conserved *Chlamydia* genus-specific B cell epitopes in the MIF antigens (13, 14, 76), the principal drawback of the C. trachomatis MIF test is high cross-reactivity of C. trachomatis EB antigens with antibodies against other chlamydial species (15–19). Additionally, it is a painstaking technique that requires extensive expertise associated with meticulous standardization of antigen preparations and subjective microscopic observation of immunofluorescence, thereby resulting in high interlaboratory variation (4, 19, 20).

In simple enzyme-linked immunosorbent assay (ELISA) format, C. trachomatis-specific serological assays are problematic due to high cross-reactivity of EBs as ELISA antigens and unavailability of suitable alternative specific antigens for sensitive detection of anti-C. trachomatis antibodies (21). Lipopolysaccharide (LPS) present on EBs is genus specific and provides substantial cross-reactivity with antibodies against other chlamydial species (14). The major constituent antigen of chlamydial EBs is the major outer membrane protein, or outer membrane protein A (MOMP/OmpA), which harbors both *Chlamydia* species-specific and genus-specific B cell epitopes, again resulting in cross-reactivity (13, 14, 22, 23). Use of other immunodominant protein antigens such as OmcB, HSP60, HSP70, HSP10, MIP, and CrpA also raises concerns about cross-reactivity, given the high sequence conservation of these proteins in *Chlamydia* spp. (21–23). The recently preferred Pgp3 C. trachomatis plasmid protein (24–28) is present and highly conserved in most other *Chlamydia* spp. infecting animal hosts (29, 30) and thus cannot resolve cross-reactivity concerns after human exposure to these animal chlamydiae, while it also may be absent in certain C. trachomatis strains following the loss of the plasmid (31, 32).

Several peptide-based ELISAs are commercially available for detection of antibodies against C. trachomatis. However, most of these peptide-based assays use single OmpA peptides or a mixture of C. trachomatis strain-specific peptides derived from polymorphic OmpA regions (33–37). Although such assays provide highly specific detection of anti-C. trachomatis antibodies when adjusted for maximum stringency, they have not received wide acceptance, presumably due to the resultant low sensitivity (38–40). Recently, several groups have used peptide antigens from other immunodominant C. trachomatis proteins (41, 42). Again, however, these assays suffer from low sensitivities.

Previously, we have identified species-specific immunodominant B cell epitopes of 11 *Chlamydia* spp. by use of *Chlamydia-*monospecific mouse hyperimmune sera raised by inoculation of live bacteria (43, 77). These peptide antigens are derived from strongly reactive regions of chlamydial immunodominant proteins (44, 45). They are sufficiently divergent to eliminate virtually any cross-reactivity with antibodies against noncognate *Chlamydia* spp. and were used as peptide antigens in ELISAs for species-specific detection of anti-*Chlamydia* antibodies (43, 77). Importantly, we showed that use of 16-mer or longer peptide antigens in an optimized ELISA format vastly increased specific signals compared to use of short peptide antigens (46). In addition to our previous identification of 10 mouse C. trachomatis peptides (43), we recently identified 38 human host-specific peptide antigens from 21 immunodominant C. trachomatis proteins, using sera of women with active C. trachomatis infections (77). Finally, we validated the utility of such peptide antigens for highly similar species- and strain-specific molecular serologies of the 11 chlamydial species spotted on peptide microarrays (47).

Given the stochastic nature of antibody responses against individual proteins (45) or B cell epitopes (43, 77), we hypothesized that measurement of combinations of antibody responses against multiple B cell epitopes of different immunodominant proteins would provide higher assay sensitivity than the use of single B cell epitope antigens, e.g., OmpA peptides. Therefore, in this study we aimed to comprehensively evaluate combinations of the top-ranked 11 C. trachomatis peptide antigens for C. trachomatis-specific detection of human antibodies. To this end, we used a well-characterized set of positive and negative-control sera and compared the peptide ELISA results for these sera to those obtained with the MIF test and 4 commercial anti-C. trachomatis IgG ELISAs. The results of the comparisons confirm profound sensitivity improvements due to the combined use of multiple highly specific peptide antigens. Thus, we present a simple ELISA methodology with defined synthetic antigens for C. trachomatis antibody detection that has the advantage of simultaneously higher sensitivity and higher specificity than standard assays.

## RESULTS

### Anti-C. trachomatis IgG antibodies determined by commercial ELISAs in sera of 125 women with active C. trachomatis infection.

Initially, the sera of 125 women with nucleic acid amplification test (NAAT)-proven active C. trachomatis infection were tested for anti-C. trachomatis IgG with four commercial ELISAs. These ELISAs use purified elementary bodies or an OmpA protein segment or OmpA peptides. Anti-C. trachomatis IgG was detected in 61% (GenWay), 58% (Serion), 53% (Savyon), and 42% (Medac) of these sera, respectively, at manufacturer-defined ELISA cutoff values (Table 1). Despite the presence of an active infection, 32% of the sera were negative for anti-C. trachomatis IgG in all 4 ELISAs tested. This incongruent outcome may have been caused by an actual absence of an antibody response against C. trachomatis in certain individuals and/or by low sensitivity of the antibody detection assays.

**TABLE 1:** Sensitivity of four commercial ELISAs for detection of anti-C. trachomatis IgG antibodies in serum of women infected with C. trachomatis^a^

Antibody assay	Serum IgG-positive frequency, %
Elementary body ELISA (GenWay)^b^	61
OmpA protein ELISA (Serion)	58
OmpA peptide ELISA (Savyon)	53
OmpA peptide ELISA (Medac)	42
	
Consensus^c^ of 4 commercial ELISAs	68

a The 125 serum samples from 125 women with active (NAAT-positive) C. trachomatis infections were included.

b The cutoff value for positivity for anti-C. trachomatis IgG antibodies was determined as recommended by the manufacturers of the ELISA kits. Borderline test results were considered negative.

c Serum samples were considered positive if the test result of any of the 4 commercial ELISA was positive.

### Antibodies against C. trachomatis-specific peptide antigens.

**FIG 1:**
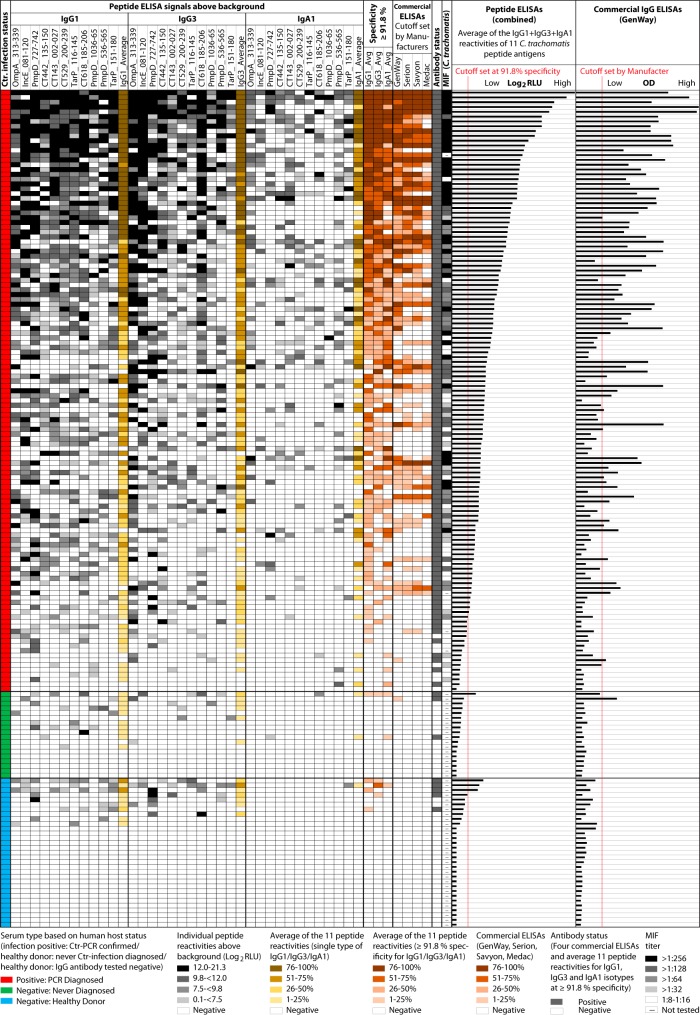
IgG1, IgG3, and IgA1 antibody reactivities of 174 human sera with 11 C. trachomatis peptide antigens. The first column indicates the origin of 125 C. trachomatis (Ctr)-positive sera of women with active C. trachomatis infection (red), 18 sera of women never diagnosed with C. trachomatis infection (green), and 31 sera of healthy female blood donors (blue). The next columns show background corrected signals with 11 individual peptides for each of three monoclonal antibody conjugates against human IgG1, IgG3, and IgA1, with reaction strength indicated by shading from white (nonreactive) to black (maximally reactive). The 11 columns corresponding to each antibody isotype are followed by data corresponding to average reactivity, indicated in quartile reactivity ranking (light to dark ochre) or as nonreactive (white). Following the columns indicating background corrected total peptide reactivities are 3 columns in white to dark brown that indicate peptide reactivity ranking at population-dependent cutoff values (91.8% specificity for IgG1 and IgG3, 93.9% specificity for IgA1). The following 4 columns indicate reactivity ranking of 4 commercial ELISAs for IgG antibody determination using manufacturer-recommended cutoff values. Next, consensus anti-C. trachomatis antibody status is shown with data representing classification of any serum as positive that was positive in any of the preceding 7 columns. The last column indicates the MIF titer, if known, with MIF-untested sera indicated by dashes. The final two bar graphs show the average IgG1 plus IgG3 plus IgA1 reactivity of each serum sample with 11 peptide antigens or with the best-performing commercial anti-C. trachomatis IgG ELISA (GenWay).

Next, sera of the 125 C. trachomatis-infected (positive) women and 49 control sera of C. trachomatis-non-infected (negative) women were tested with ELISAs using 11 C. trachomatis peptide antigens (Table 2) and horseradish peroxidase (HRP) conjugates against human IgG1, IgG3, and IgA1 (Fig. 1). IgG1 and IgG3 antibodies dominated the responses to individual peptide antigens (B cell epitopes). However, the response pattern was stochastic for all antibody isotypes (48), and even sera that reacted strongly overall showed holes in the peptide reactivity patterns (Fig. 1).

**TABLE 2:** C. trachomatis peptide antigens used in this study

Peptide antigen	Peptide sequence^a^	Avg % sequence conservation within C. trachomatis^b^	Avg probability of reactivity within C. trachomatis^c^	Maximum probability of cross-reactivity with^d^:		
				C. suis or C. muridarum	C. pneumoniae	Chlamydia spp.
CtrOmpA_313-339	IFDTTTLNPTIAGAGDVKTGAEGQLGD	74	0.42	0.55	—	—
CtrIncE_81-120	LFAISALDVLEDHGLVGCPFKLPCKSSPANEPTVQFFKGK	97	0.93	0.02	—	—
CtrPmpD_727-742	EKVEEVEPAPEQKDNN	100	0.95	—	—	—
CtrCT442_135-150	VVESLSRRNSLVDQTQ	99	0.94	—	—	—
CtrCT143_2-27	KKPVFTGGAPIPGISTEEGTGVKDQN	100	0.95	0.20	—	—
CtrCT529_200-239	SAERADCEARCARIAREESLLEVPGEENACEKKVAGEKAK	96	0.92	—	—	—
CtrTarP_116-145	TSSSDHIPSDYDDVGSNSGDISNNYDDVGS	77	0.50	—	—	—
CtrCT618_185-206	GNLKQNKPTEGTSKENGFMARL	99	0.94	—	—	—
CtrPmpD_1036-65	SGTPVQQGHAISKPEAEIESSSEPEGAHSL	98	0.94	—	—	—
CtrPmpD_536-565	ARAPQALPTQEEFPLFSKKEGRPLSSGYSG	100	0.95	—	—	—
CtrTarP_151-180	SSNYDDAAADYEPIRTTENIYESIGGSRTS	95	0.91	—	—	—

a Only the actual C. trachomatis serovar D strain D/UW-3/Cx peptide antigen sequences are shown, without the N-terminal biotin and serine-glycine-serine-glycine spacer that is attached to each peptide (43).

b Average percent amino acid sequence identity with 22 C. trachomatis strains representing all major C. trachomatis clades is shown. Ctr, C. trachomatis; Csu, C. suis; Cmu, C. muridarum; Cpn, C. pneumoniae; *Chlamydia* spp., the remaining species of the genus *Chlamydia* (i.e., those other than C. trachomatis, C. suis, C. muridarum, and C. pneumoniae).

c Average probability of reactivity with sera raised against other *C. trachomatis* serovars is shown, based on calculation by peptide sequence conservation (43).

d Probability of cross-reactivity is shown with sera specific for C. muridarum or C. suis, the two species that are phylogenetically closest to C. trachomatis. Dashes indicate ≤0.01 probability of cross-reactivity.

### Anti-C. trachomatis IgG1, IgG3, and IgA1 antibody frequencies in women with active C. trachomatis infection.

For each of three isotypes, the average signal (in Log_2_ relative light units [RLU]) of the 11 C. trachomatis peptide antigens (Table 2) for the 49 C. trachomatis-negative-control sera was used to define cutoff values. At 91.8% (IgG1), 91.8% (IgG3), and 93.9% (IgA1) specificity cutoff values, 80.8%, 69.6%, and 68.8% of sera from C. trachomatis*-*infected women were positive for IgG1, IgG3, and IgA1 antibodies, respectively (Fig. 1). When the 3 antibody isotype-specific signals (IgG1, IgG3, and IgA1) for the 11 C. trachomatis peptides were averaged and 85%, 90%, and 95% specificity cutoff values were chosen, the detection frequencies (assay sensitivity) of anti-C. trachomatis antibodies in C. trachomatis-infected women were 90.4%, 85.6%, and 75.8% (Table 3). The IgG assay (IgG1 plus IgG3) at the same specificity cutoff values achieved 88.6%, 83.2%, and 72.7% sensitivities (Table 3). These sensitivities were higher by 27.8%, 22.4%, and 11.9%, respectively, than the sensitivity of the GenWay IgG ELISA, the best commercial test (Table 1) (*P* = <10^−5^, <10^−4^, and <0.06, respectively [Fisher's exact test]).

**TABLE 3:** Prediction of C. trachomatis infection status by seroreactivities of peptide antigens determined by ROC curve analysis^a^

Avg reactivity of 11 C. trachomatis peptides^b^	% sensitivity			AUC ± SEM
	Spec = 85%	Spec = 90%	Spec = 95%	
IgG1 + IgG3 + IgA1	90.4	85.6	75.8	0.951 ± 0.017
IgG1 + IgG3	88.6	83.2	72.7	0.944 ± 0.018
IgG3 + IgA1	85.5	79.0	66.6	0.931 ± 0.021

a The 125 C. trachomatis infection-positive and 49 C. trachomatis infection-negative sera were used as categorical positive/negative variables known *a priori*, and the observed values of serum reactivities of a test were used as predictor variables of the C. trachomatis infection status. Spec, specificity.

b Individual plasma samples were tested with each of the 11 peptide antigens by use of single IgG1, IgG3, or IgA1 detection conjugates. The reactivities of the 11 peptides were combined by averaging the reactivities in Log_2_ RLU per second for 2 or all 3 conjugates.

### Performance of individual assays compared to consensus for anti-C. trachomatis antibody status.

A binomial consensus antibody status was established for all 174 serum samples, with any serum considered positive if it was positive in any 1 of the 4 commercial ELISAs or for peptide reactivity with any 1 of the 3 antibody isotypes (Fig. 1). Based on this consensus, 126 sera were classified as positive and 48 sera as negative for anti-C. trachomatis antibodies (Fig. 1). Using the consensus as the gold standard for anti-C. trachomatis antibody detection, the sensitivities of individual assays at different specificity cutoff values were calculated from receiver operating characteristic (ROC) curves (Table 4). For example, the sensitivities of commercial ELISAs at 98% specificity cutoff values were 64.8% for GenWay, 52.8% for Serion, 52.9% for Savyon, and 51.5% for Medac (Table 4). Using the combined average of optical density (OD) values of these four assays, the sensitivity at the 98% specificity cutoff increased marginally to 66.9% (Table 4). In contrast, the combined average of IgG1 plus IgG3 plus IgA1 ELISA signals for 11 C. trachomatis peptide antigens achieved 92.9% sensitivity at the same 98% specificity cutoff and 91.8% sensitivity with only IgG1 plus IgG3 conjugates. This level of assay performance is significantly higher than that of any commercial ELISA (*P* < 10^−5^; Fisher's exact test). However, IgG3 plus IgA1 peptide reactivity achieved only 77.2% sensitivity compared to 91.8% sensitivity achieved by IgG1 plus IgG3. These data indicate that the level of the unique contribution of the long-lived IgG1 isotype to the short-lived IgG3 isotype is higher than that of IgA1, another short-lived isotype (*P* = 0.002; Fisher's exact test). At a less stringent cutoff such as 90% specificity, the sensitivity of all peptide assays increased. This was most pronounced for IgG3 plus IgA1, which increased in sensitivity by 16% from 77.2% to 93.2% (Table 4), while IgG1 plus IgG3 sensitivity increased by only 3.9% from 91.8% to 95.7%.

**TABLE 4:** Sensitivity for determination of anti-C. trachomatis antibody status at different assay specificities determined by ROC curve analysis^a^

Antibody assay	% sensitivity^b^					Avg % sensitivity at specificity of 98%–80%^c^	AUC ± SEM
	Spec = 80%	Spec = 85%	Spec = 90%	Spec = 95%	Spec = 98%		
Peptides (IgG1 + IgG3 + IgA1)^d^	98.0	97.5	96.8	95.2	92.9	96.1	0.987 ± 0.007
Peptides (IgG1 + IgG3)	97.2	96.6	95.7	94.1	91.8	95.1	0.981 ± 0.009
Peptides (IgG3 + IgA1)	97.3	95.8	93.2	87.3	77.2	90.2	0.975 ± 0.011
							
GenWay ELISA	85.1	82.5	78.8	72.6	64.8	76.8	0.906 ± 0.022
Serion ELISA	76.2	72.9	68.4	61.2	52.8	66.3	0.847 ± 0.029
Savyon ELISA	80.4	76.8	71.6	63.2	52.9	69.0	0.880 ± 0.026
Medac ELISA	81.9	78.0	72.4	63.0	51.5	69.4	0.891 ± 0.025
							
Avg OD value of the four commercial ELISAs	84.9	82.5	79.3	73.8	66.9	77.5	0.903 ± 0.022
							
Single CtrOmpA_313-339 peptide (IgG1 + IgG3)	87.2	84.4	80.4	73.4	64.3	77.9	0.921 ± 0.035

a Individual assays were compared to the C. trachomatis antibody consensus. The consensus for the C. trachomatis antibody status was derived by classifying any serum as antibody positive if the test result for any 1 of 7 tests was positive. These tests were the 4 commercial ELISA and the IgG1, IgG3, or IgA1 ELISAs combining the 11 C. trachomatis peptide antigens.

b Sensitivity means of the combined peptide reactivity assays were highly significantly higher than those determined for any of the four commercial ELISAs or for the peptide assay using the single OmpA antigen CtrOmpA_313-339 (*P* ≤ 0.003; Student’s *t* test). The CtrOmpA_313-339 assay with IgG (IgG1 plus IgG3) detection was significantly more sensitive than any OmpA antigen-based ELISA (Serion, Savyon, or Medac; *P* ≤ 0.002).

c Average sensitivity was calculated from the sensitivities at 5 different assay specificities (98%, 95%, 90%, 85%, and 80%).

d Average of IgG1 plus IgG3 plus IgA1 reactivities for 11 C. trachomatis peptides.

### Concordance of total peptide reactivity with commercial ELISAs.

**FIG 2:**
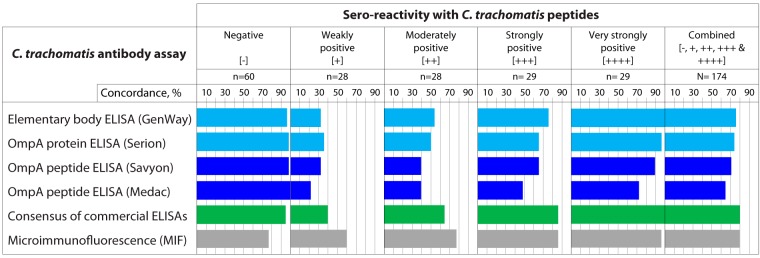
Concordance of C. trachomatis peptide reactivity assay with commercial ELISAs. The reactivity of each serum with 11 C. trachomatis peptide antigens was calculated by averaging IgG1 plus IgG3 plus IgA1 Log_2_ RLU signals. A cutoff value was chosen at 91.8% specificity such that among the 49 C. trachomatis-negative sera, maximally 4 sera were falsely classified as positive for C. trachomatis antibodies. Of 174 sera, the 114 peptide-reactive sera were scored +1 to +4 based on their reactivity rank. Evaluation of MIF concordance used only the 121 sera with known MIF titer values (Fig. 1). Peptide reactivity showed significantly higher concordance with the GenWay and Serion ELISAs than with the Medac ELISA (*P* ≤ 0.047; Fisher's exact test).

For each serum, the IgG1 plus IgG3 plus IgA1 signals of all 11 C. trachomatis peptides were averaged and a 91.8% specificity cutoff value was chosen such that 45 of 49 C. trachomatis-negative sera were classified as negative (Fig. 1). At this cutoff, 110 (88%) of 125 sera of women with C. trachomatis infection were positive for anti-C. trachomatis antibodies. The average concordance between the very strongly peptide-reactive and peptide-negative sera and the four commercial ELISAs was high (89.7% and 98.3%, respectively; Fig. 2), indicating highly specific reactivity of C. trachomatis peptide antigens. In contrast, the majority of discordant results were observed for sera with low peptide reactivity, which were frequently negative in the commercial ELISAs (Fig. 2), most likely due to poor ELISA sensitivity. Overall, the average levels of seroreactivity with C. trachomatis peptide antigens were 76.5% in concordance for GenWay, 74.7% for Serion, 71.3% for Savyon, and 64.4% for Medac ELISAs. The significantly higher concordance of the EB antigen-based GenWay ELISA than of the OmpA peptide-based Savyon and Medac ELISAs (*P* ≤ 0.047; Fisher's exact test) may have been related to the complexity of the EB antigen, whose reactivity resembles the reactivities of 11 peptide epitopes of 8 immunodominant proteins (Table 2).

### Predictive power of single versus combined peptide antigens.

Use of the area under the ROC curve (ROC-AUC) as a global assay performance measure (Table 4) indicated that the assay using the IgG1 plus IgG3 plus IgA1 reactivity average of 11 C. trachomatis peptide antigens (AUC = 0.987) significantly outperformed any of the 4 commercial ELISAs (maximum AUC = 0.906 for GenWay; *P* = 0.001). (An AUC value of 0.50 indicates that discrimination of positive data from negative data equals random categorization [no discrimination], and an AUC value of 1.0 indicates that prediction is perfect [100% accurate discrimination]). This was, however, achieved by 33 individual tests of a single serum (3 antibody isotypes × 11 peptide antigens). To reduce this extensive number of assays, we used ROC-AUC analyses to identify the minimal combination of peptide antigens that still provided high assay performance. As shown in Table 5, the diagnostic performance of individual peptide antigens varied substantially, but the relative ranking results were similar irrespective of the detected binding antibody isotype. Compared to the best-performing single peptide, addition of even one peptide to achieve a combination of two peptide assays improved performance dramatically. For example, the AUC_IgG1+IgG3+IgA1_ of peptide 1 increased from 0.889 to 0.947 when peptide 2 was added. Further stepwise addition of peptide antigens in ROC analyses showed that beyond the combination of the 5 top peptide antigens (AUC_IgG1+IgG3+IgA1_ = 0.979), addition of any one or more of the remaining 6 peptides improved the combined assay only marginally (AUC_IgG1+IgG3+IgA1_ = 0.987), with similar trends for all detected antibody isotypes (Table 5).

**TABLE 5:** Predictive power (ROC-AUC) of single or multiple peptide antigens and antibody isotypes for determination of anti-C. trachomatis antibody status^a^

Peptide(s)^b^	ROC-AUC value					
	IgG1	IgG3	IgA1	IgG1 + IgG3^c^	IgG3 + IgA1	IgG1 + IgG3 + IgA1
1	0.776	0.836	0.643	0.884	0.849	0.889
2	0.803	0.762	0.583	0.879	0.774	0.891
3	0.730	0.706	0.583	0.792	0.737	0.812
4	0.754	0.770	0.615	0.849	0.800	0.869
5	0.796	0.696	0.631	0.859	0.757	0.876
6	0.800	0.651	0.599	0.838	0.699	0.854
7	0.778	0.679	0.552	0.822	0.698	0.823
8	0.733	0.759	0.607	0.831	0.778	0.849
9	0.696	0.651	0.528	0.745	0.656	0.749
10	0.666	0.683	0.659	0.756	0.763	0.798
11	0.739	0.578	0.615	0.748	0.650	0.773
1–2	0.875	0.900	0.687	0.942	0.904	0.947
1–3	0.876	0.917	0.718	0.948	0.938	0.959
1–4	0.903	0.934	0.750	0.964	0.954	0.971
1–5	0.913	0.940	0.774	0.975	0.960	0.979
1–6	0.918	0.941	0.794	0.978	0.960	0.983
1–7	0.914	0.946	0.798	0.977	0.966	0.981
1–8	0.913	0.944	0.810	0.979	0.964	0.982
1–9	0.913	0.944	0.810	0.980	0.963	0.983
1–10	0.915	0.947	0.841	0.980	0.970	0.987
1–11	0.919	0.945	0.853	0.982	0.970	0.987

a The antibody consensus of 125 C. trachomatis infection-positive and 49 C. trachomatis infection-negative sera was used as a categorical variable known *a priori* (Fig. 1), and the observed values of peptide reactivities (Log_2_ RLU signals) were used as predictor variables. Area under the ROC curve (AUC) analyses were performed as described in Materials and Methods.

b Peptide 1, CtrOmpA_313-339; peptide 2, CtrIncE_81-120; peptide 3, CtrPmpD_727-742; peptide 4, CtrCT442_135-150; peptide 5, CtrCT143_2-27; peptide 6, CtrCT529_200-239; peptide 7, CtrTarP_116-145; peptide 8, CtrCT618_185-206; peptide 9, CtrPmpD_1036-65; peptide 10, CtrPmpD_536-565; peptide 11, CtrTarP_151-180. Paired numbers correspond to peptide combinations for the multipeptide ELISAs, where "1–2" indicates that the reactivities of peptides 1 and 2 were averaged, "1–3" indicates that the reactivities of peptides 1, 2, and 3 were averaged, etc., for each detection with one to three conjugates.

c Individual plasma samples were tested with each of the 11 peptide antigens by use of single IgG1, IgG3, or IgA1 detection conjugates. The reactivities were combined by averaging the reactivities (Log_2_ RLU per second) for 2 or 3 conjugates.

### Comparative performance of an optimum IgG assay with 5 C. trachomatis-specific peptide antigens.

**FIG 3:**
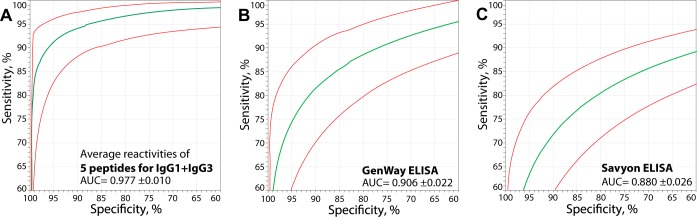
ROC evaluation of individual anti-C. trachomatis IgG assays using the consensus antibody status as the gold standard. The antibody consensus of 125 C. trachomatis infection-positive and 49 C. trachomatis infection-negative sera was used as the categorical variable known *a priori*, and the observed values of serum reactivities of a test were used as predictor variables of the anti-C. trachomatis antibody status. (A) The average Log_2_ RLU signals of the first 5 strongly reactive peptide antigens for IgG1 plus IgG3 conjugates (Table 5) were used as predictor variables. The solid green line indicates the maximum likelihood-fitted ROC curve, and red lines indicate the 95% CI. (B and C) The OD values were used as predictor variables for commercial ELISAs. The average sensitivity of IgG1 plus IgG3 detection of five peptides (92.7%) at a given set of specificities (98%, 95%, 90%, 85%, and 80%) was significantly higher than the average sensitivities obtained with commercial GenWay (76.7%) (B) and Savyon (69.0%) (C) ELISAs (*P* ≤ 0.0008; Student’s paired *t* test).

For sensitive detection of anti-C. trachomatis antibodies with a reduced number of tests, the ROC curve of an optimum IgG1 plus IgG3 assay with 5 peptide antigens was determined (Fig. 3A). The IgG1 plus IgG3 reactivity average for 5 C. trachomatis peptide antigens achieved a 92.7% average sensitivity for 98%, 95%, 90%, 85%, and 80% specificities (Fig. 3A). In contrast, at the same specificity cutoff values, the 4 commercial ELISAs achieved significantly lower sensitivities of 76.7% (GenWay), 66.3% (Serion), 69.0% (Savyon), and 69.4% (Medac) (Table 4 and Fig. 3B and C) (*P* ≤ 0.0008; one-tailed paired Student’s *t* test). Thus, an assay for IgG1 plus IgG3 detection performed with just 5 C. trachomatis peptide antigens achieved substantially higher (16.0% to 26.4% increase) sensitivity than any of 4 commercial ELISAs at the same set of specificity cutoff values.

### Diagnostic suitability of assays determined by likelihood ratios.

**FIG 4:**
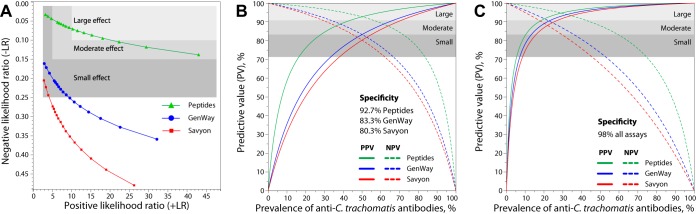
Diagnostic utility modeling of anti-C. trachomatis antibody assays by likelihood ratios and predictive values. For the peptide assay, adequate performance at reasonable levels of laboratory effort was chosen by using the 5 top-performing peptides and IgG1 and IgG3 conjugates (Table 5) (Fig. 3A). (A) Positive and negative likelihood ratios are independent of antibody prevalence. Sensitivities were calculated at specificities ranging from 70% (left) to 98% (right). Using sensitivity and specificity data of ROC curves (Fig. 3), positive-likelihood ratios (+LR) and negative-likelihood ratios (−LR) were calculated. The best performance of an assay is found at high +LR and low −LR (top right). At +LR from 5 to 25, the 0.082 −LR average of peptide reactivity differs highly significantly from the 0.279 −LR of the GenWay ELISA or the 0.391 −LR of the Savyon ELISA (*P* < 10^−3^; paired Student’s *t* test). (B and C) Positive and negative predictive values in dependence on the prevalence of populations of anti-C. trachomatis antibodies. Sensitivity cutoff values and the resultant specificities in ROC curves (Fig. 3) were selected for high (B) and low (C) prevalence. (B) When equal sensitivity and specificity levels of an assay are chosen from the ROC curve, PPV and NPV of the assay become equal at an assumed 50% antibody prevalence. Importantly, the peptide reactivity assay achieved higher performance (sensitivity = specificity = PPV = NPV = 92.7%) than the GenWay (83.3%) and Savyon (80.3%) ELISAs. The 3-Gy shaded areas in panel B indicate the zones of high, moderate, and poor performance of the assays, corresponding to large, moderate, and small diagnostic effects (both PPV and NPV were ≥90.9%, 83.3%, and 71.4%, respectively). For high performance of the peptide assay, a high (≥90.9%) PPV is obtained in the 44% to 100% prevalence range and a high (≥90.9%) NPV in the 0% to 56% range. This creates an overlapping 44% to 56% prevalence range in which the peptide assay delivers high PPV as well as NPV values (high performance) and a 29% to 71% prevalence range that delivers moderate performance. None of the commercial ELISAs delivered high or moderate performance for both PPV and NPV, and GenWay and Savyon delivered only poor performance at prevalence ranges of 34% to 66% (GenWay) and 38% to 62% (Savyon). At 38% to 62% prevalence, the PPV and NPV values of the peptide assay differed highly significantly from those of the GenWay or Savyon ELISAs (*P* < 0.001; paired Student’s *t* test). (C) Modeled for lower antibody prevalence, 98% specificity was chosen for all assays. The peptide assay delivers high and moderate performance for prevalence ranges of 19% to 42% and 11% to 59%, respectively. In contrast, the commercial ELISAs at best achieved moderate performance at 14% to 35% prevalence (GenWay) and at 16% to 29% prevalence (Savyon). At 14% to 35% prevalence, the peptide reactivity assay performed highly significantly better than any of the four commercial ELISAs (*P* < 0.002; paired Student’s *t* test).

Next, we sought to comparatively assess the diagnostic utility of the C. trachomatis peptide IgG assay and the commercial anti-C. trachomatis IgG ELISAs by use of likelihood ratios (LR). For the assay with 5 C. trachomatis peptide antigens and IgG1 plus IgG3 detection (Fig. 4A), any 91% to 96% specificity cutoff value corresponding to ~94% to 90% sensitivities (Fig. 3A) achieved large diagnostic effect sizes for both positive-likelihood ratios (+LR = 10.4 to 22.5) and negative likelihood ratios (−LR = 0.07 to 0.10). In contrast, for the best-performing GenWay ELISA (Fig. 4A), cutoff values at 70% to 90% specificity with corresponding ~89% to 77% sensitivities (Fig. 3B) achieved only small diagnostic effect sizes for both +LR (3.0 to 9.6) and −LR (0.16 to 0.25). At +LR from 5 to 25 (Fig. 4A), the −LR average of peptide reactivity (0.082) was significantly lower (*P* < 10^−3^) than the −LR of the GenWay ELISA (0.279). The level of performance of the Savyon ELISA (Fig. 4A) as representative of remaining 3 commercial ELISAs was even lower than that of the GenWay ELISA.

### Diagnostic suitability of assays determined by predictive value modeling in dependence on anti-C. trachomatis prevalence.

Finally, we used predictive value modeling to comparatively assess the diagnostic utility of the C. trachomatis peptide IgG assay (IgG1 plus IgG3) and the commercial anti-C. trachomatis IgG ELISAs in populations with different anti-C. trachomatis antibody prevalences. For the C. trachomatis peptide IgG assay set at 92.7% specificity/sensitivity, high performance (positive predictive value [PPV] and negative predictive value [NPV] ≥ 90.9%) and moderate performance (PPV and NPV ≥ 83.3%) were obtained at prevalence ranges of 44% to 56% and 29% to 71%, respectively (Fig. 4B). For the best commercial ELISAs, only a significantly smaller (*P* < 0.001) diagnostic effect size (poor performance; PPV and NPV ≥ 71.4%) was obtained for a prevalence range of 34% to 66% (GenWay) or 38% to 62% (Savyon) (Fig. 4B).

For low-prevalence populations, the C. trachomatis peptide IgG assay (IgG1 plus IgG3) achieved high and moderate performance for prevalence ranges of 19% to 42% and 11% to 59%, respectively. In contrast, the commercial ELISAs at best achieved significantly reduced (*P* < 0.002), moderate performance at 14% to 35% (GenWay) and 16% to 29% prevalence (Savyon) (Fig. 4C). Note that the low sensitivity of the commercial ELISAs resulted in a precipitous NPV drop when a high-specificity cutoff value (and PPV) was chosen.

### Diagnostic usefulness of assays using 11 versus 5 C. trachomatis peptides.

Depending on the intended use of anti-C. trachomatis antibody data, a minimal set of the most informative peptide antigens combined with detection of one or two antibody isotypes may reduce the required number of tests substantially without compromising performance (Table 5). Therefore, we compared several minimal assays with the full assay that used all 11 C. trachomatis peptide antigens and conjugates against three antibody isotypes (see Fig. S1 in the supplemental material). ROC curves, +LR, −LR, PPV, and NPV were evaluated similarly to the methods described for Fig. 3 and 4 to determine suitable assay cutoff values (Fig. S1). As reported in Table 5, any reduction of either peptide antigen numbers or detected antibody isotypes reduced assay performance, particularly at high-stringency cutoff values. For instance, at a 98% specificity cutoff, the IgG1 plus IgG3 plus IgA1 reactivity average of 11 peptides achieved 92.9% sensitivity whereas the IgG1 plus IgG3 reactivity average of 5 peptide antigens achieved only 86.5% sensitivity, a 7.4% sensitivity reduction (Fig. S1A). Thus, the best assay performance was obtained with the maximum number of 11 peptide antigens and detection of all three antibody isotypes, but use of 5 optimal peptides still achieved adequate diagnostic performance (Fig. S1B and C and D). The data shown in Table 5 (see also Fig. S1) will help medical practitioners to choose the combination of peptide antigens and conjugates most appropriate for specific epidemiological settings.

10.1128/mSphere.00253-18.1 FIG S1  Utility of C. trachomatis peptide antigen assays evaluated by ROC curves, likelihood ratios, and predictive values. Performance differences between C. trachomatis peptide antigen assays were determined using the complete set of 11 peptides and the IgG1 plus IgG3 plus IgA1 conjugate combination versus assays with the reduced set of the 5 most informative peptides and different conjugate combinations. The antibody consensus of 125 C. trachomatis infection-positive and 49 C. trachomatis infection-negative sera was used as the categorical variable known *a priori*, and the observed values of serum reactivities of a test were used as predictor variables of the anti-C. trachomatis antibody status. The average Log_2_ RLU signals of the peptide antigens for different combinations of conjugates were used as predictor variables. For reference, the green lines indicating the 5-peptide assay with IgG1 plus IgG3 detection correspond to those in Fig. 3 and 4. (A) ROC evaluation of individual anti-C. trachomatis antibody assays against the gold standard consensus antibody status. Solid color lines indicate maximum likelihood-fitted ROC curves. The dotted lines indicate sensitivity at 98% specificity. (B) Positive-likelihood and negative-likelihood ratios are independent of the population prevalence of anti-C. trachomatis antibodies. Sensitivities were calculated at specificities ranging from 70% (left) to 90% (right). Using sensitivity and specificity data, positive-likelihood ratios (+LR) and negative-likelihood ratios (−LR) were calculated. (C and D) Positive and negative predictive values in dependence of anti-C. trachomatis antibody population prevalence. (C) PPV, NPV, sensitivity, and specificity were equal for each assay at an assumed high 50% antibody prevalence but were higher for the IgG1 plus IgG3 plus IgA1 reactivities of 11 peptide antigens (PPV = NPV = sensitivity = specificity = 95.1%) than for the conjugate combinations with 5 peptides. (D) In modeling for conditions of lower antibody prevalence, 98% specificity was chosen for all assays. The assay with 11 peptides and 3 conjugates also showed the highest performance. Download FIG S1, PDF file, 0.7 MB. Copyright © 2018 Rahman et al. 2018 Rahman et al. This content is distributed under the terms of the Creative Commons Attribution 4.0 International license.

## DISCUSSION

We demonstrate here that peptides of C. trachomatis-specific B cell epitopes, in combined use, are suitable ELISA antigens for highly sensitive and specific detection of anti-C. trachomatis antibodies. The reactivity “holes” (false-negative results) of individual peptide antigens (Fig. 1) or whole-protein antigens (45) with even strongly antibody-positive sera corroborate the contention that the use of single peptide and protein antigens would increase false-negative results. Thus, only the combined use of multiple peptide antigens can reliably quantify the host antibody level produced in response to C. trachomatis infections, approximating the quantitative results obtained with complex antigens but typically at higher specificity. For adequate performance with an acceptable workload, we recommend the use of the five strongest C. trachomatis peptide antigens as an alternative to the maximum performance obtained by use of 11 peptides (Table 5; see also Fig. S1 in the supplemental material). While many commercial anti-C. trachomatis ELISAs are exclusively based on OmpA antigens, we conclude that the higher sensitivity of the peptide assays described here is the result of the use of multiple B cell epitopes chosen from several immunodominant C. trachomatis proteins in addition to OmpA (Table 5). Therefore, multipeptide antigens capture a wide spectrum of antibodies, similarly to complex chlamydial antigens with multiple epitopes (e.g., elementary bodies), but avoid the cross-reactivity associated with conserved epitopes within such complex antigens. Furthermore, the high sensitivity is also the consequence of the peptide antigen design and the ELISA protocol optimized for maximum seroreactivity (43, 46, 47, 77). Thus, the present report offers a simple ELISA with defined synthetic antigens for C. trachomatis antibody detection that has the advantage of simultaneous high sensitivity and high specificity (Table 4).

In the absence of a gold standard, the peptide reactivity assays were evaluated using two composite reference standards (CRS). Such CRS typically consist of several imperfect tests with poor sensitivity but high specificity (49–52). Excluding the peptide reactivity assays (index tests), the NAAT and four commercial ELISAs were used as component tests to establish a first CRS for C. trachomatis exposure status (Table 3). This evaluation established the superior performance of the peptide assays for determination of C. trachomatis exposure status via use of anti-C. trachomatis antibodies. For subsequent comparative performance evaluation of all individual assays, a second CRS was constructed from 7 component tests, including 4 commercial ELISAs and tests of IgG1, IgG3, and IgA1 reactivity averages of 11 C. trachomatis peptide antigens (Table 4; termed "consensus" in Fig. 1). It is well known that increasing the numbers of component tests in CRS reduces the number of false negatives (higher sensitivity) but increases the number of false positives (lower specificity) unless all component tests have perfect specificity (49–52). For that reason, we minimized the input of peptide antigen data by using averages rather than numerous individual peptide reactivities and applied stringent cutoff values for high specificity (Fig. 1). Supporting the validity of the CRS approach, previous studies published by us as well as by others reported high specificity for C. trachomatis-specific peptide antigens (also used in Savyon, Serion, and Medac ELISAs) but poor sensitivity (41–47, 53, 54, 77). Taken together, these considerations strongly favored the use of CRS over an alternative gold standard determined by latent class analysis (LCA), because the ELISA assays in this investigation (except for NAAT) clearly violate the required assumption of the conditional independence of the LCA component tests (49–52, 55).

For useful diagnostic antibody detection, a highly desirable characteristic of an assay is that it maintains high predictive values, both positive and negative, at a wide range of antibody prevalences. For assays that provide acceptable performance only within a narrow prevalence range, signal cutoff stringency can be adjusted to an anticipated population prevalence level. This entails a tradeoff between assay specificity and sensitivity. ROC curves can be used to identify the suitable specificity/sensitivity points and associated assay signal cutoff values that result in PPV and NPV balanced for the anticipated population prevalence. For example, a stringent cutoff (high background cutoff = high specificity) resulting in high PPV is required for low population prevalences of anti-C. trachomatis antibodies (Fig. 4C; see also Fig. S1D). Therefore, a tradeoff between specificity and sensitivity must be found that maximizes both PPV and NPV for the antibody prevalence in question. As shown in Table 4 (see also Fig. S1A), for a high-stringency cutoff (98% specificity), the combined use of 5 to 11 peptide antigens still provided high sensitivity (86.5% to 91.8%; IgG1 plus IgG3 conjugates). This resulted in moderate to high performance (PPV and NPV ≥ 88%) in a wide anti-C. trachomatis antibody prevalence range of 14% to 49%. In contrast, with 64.8% sensitivity at 98% specificity, the best-performing GenWay ELISA achieved these performance characteristics only within a narrow 19% to 27% prevalence range. The remaining three commercial ELISAs did not provide even that level of performance for any prevalence (Fig. 4C), due to poor assay sensitivity (51.5% to 52.9%) at this high (98%) specificity cutoff (Table 4).

Likelihood ratios are another informative measure of assay performance in diagnostic testing. Unlike predictive values, likelihood ratios measure assay performance independently of antibody prevalence. As determined by the likelihood ratios, the multipeptide assay again outperformed the commercial ELISAs, with large diagnostic effect sizes for both +LR = ≥10 and −LR = ≤0.10 by the assays using 5 to 11 peptides for IgG (IgG1 plus IgG3) but not with any of the four commercial IgG ELISAs (Fig. 4A; see also Fig. S1B). Analyses of the area under the ROC curve (ROC-AUC), another cutoff-independent global assay characteristic, again showed higher performance for multipeptide assays than for commercial ELISAs (Table 4).

To capture the history of C. trachomatis exposure, we recommend detection of both long-lived IgG1 and short-lived IgG3 (Table 5; see also Fig. S1). For recent exposure, detection of IgG3 plus IgA1 is more appropriate, due to the immediate response and the short half-life of these antibody isotypes (37, 56, 57). Importantly, for antibody status determination by use of only two monoclonal conjugates, the IgG3 plus IgA1 combination may have an advantage for detection of recent chlamydial infections (37, 56, 57), while the IgG1 plus IgG3 combination is optimal for detection of total antibody status. While numerous combinations of peptide antigens showed virtually identical levels of performance with the study sera, we suggest use of the five top-ranked peptides (Table 5), whose performance in combination is closest to that seen with the best combination of all 11 peptides. These five immunodominant B cell epitopes of C. trachomatis OmpA, IncE, PmpD, CT143, and CT442 proteins (Table 5) are highly divergent from those of other *Chlamydia* spp. but highly conserved within C. trachomatis (Table 2). Combined with our previous studies (43, 46, 47, 77), the result of this study opens the potential for novel *Chlamydia* species serology with very high specificity at sensitivity equal to or better than what classical assays for serodiagnosis of chlamydial infection offer. The simple synthetic peptide ELISA format is within reach of any diagnostic laboratory and can be readily commercialized similarly to OmpA peptide ELISAs (33–40).

For specific detection of anti-C. trachomatis antibody, the majority of currently available commercial ELISAs use only C. trachomatis-specific OmpA peptide or recombinant protein segment antigens, thereby avoiding cross-reactivity. Those assays provide specific ELISA signals but, because of the use of the single antigen, also have unacceptably poor assay sensitivity as a consequence of failing to detect antibody responses against other immunodominant proteins of C. trachomatis. Several studies reported that C. trachomatis-positive human sera recognize non-OmpA proteins such as polymorphic outer membrane proteins (Pmp) and inclusion membrane proteins (Inc) equally frequently or even more frequently (44, 45). Importantly, using a C. trachomatis proteome-wide microarray, Wang et al. reported that antibodies to OmpA are present in only 58% of anti-C. trachomatis-positive sera, although high levels of antibodies to other C. trachomatis proteins are detectable in the remaining 42% (45). Such false-negative results may be a consequence of well-known problems with MOMP expression and epitope “masking” in incorrectly folded recombinant proteins or of the randomness of somatic recombination of the antigen recognition regions of immunoglobulin genes (48). Thus, the reactivity “holes” (false-negative results) of even highly antibody-positive sera (Fig. 1) unequivocally imply that use of single antigens in serology inherently compromises sensitivity as well as quantitative accuracy.

Intact chlamydial elementary body antigens serve as antigen in the gold-standard MIF test for anti-C. trachomatis-specific antibody detection, and EB or EB lysates are also commonly used in ELISAs. In these complex antigens, OmpA is the primary reactant for species-specific anti-C. trachomatis antibodies, but genus-specific LPS (14) or conserved B cell epitopes on CT681/OmpA, CT443/OmcB/Omp2, CT442/CrpA, Hsp60, or CT381/ArtJ frequently cross-react with antibodies against other chlamydial species, including C. pneumoniae (13, 22, 23, 58). A Pgp3 protein-based ELISA that was recently used extensively for *C. trachomatis* serology showed 11% to 14% higher sensitivity than the OmpA peptide-based Savyon and Medac ELISAs (40). In contrast, the IgG1 plus IgG3 peptide assay reported here showed ~40% higher sensitivity than the Medac and Savyon ELISAs, at similar (98%) specificity (Table 4). Therefore, both the inherently low specificity of assays using complex cross-reactive antigens and the inherently low sensitivity of single-protein assays impede their use in control and monitoring programs for trachoma and sexually transmitted C. trachomatis infections (9, 40, 59–63).

In a previous study, we reported 48 C. trachomatis-specific peptide antigens; we comprehensively evaluated the top 11 of those peptides in the present study. Using four of those peptides modified for a microarray, we recently showed that these peptide antigens similarly detect anti-C. trachomatis antibodies in microarray format with higher sensitivity than a commercial ELISA. Serological assays for reliable measurement of antibody levels with high sensitivity require the use of multiple peptide antigens but also a setup using single peptides per microtiter well. Therefore, multiple wells must be used for each individual serum sample, incurring a substantial workload. Microarray chips can incorporate dozens of peptide antigens, thus eliminating the numerical and labor constraints associated with multipeptide microtiter plate ELISAs. Therefore, the logical use of these peptide antigens in real-life implementation of *C. trachomatis* serology is in multiplexed microarray format (47, 64, 65). With the possibility of spotting 196 to 784 peptides on a single microarray chip, such microarrays offer unprecedented opportunities (65). Combined multipeptide signals not only would allow specific and accurate quantification of anti-C. trachomatis antibody responses but also might reveal multiepitope reaction patterns (Fig. 1) that may correlate with disease phenotypes and thus may serve as disease biomarkers (61–63, 66–70).

Seroepidemiology based on highly specific and sensitive assays can determine past exposure and measure the cumulative risk of C. trachomatis infection over time (4–7) and thus can help to minimize and control the burden of C. trachomatis infections in populations (71). The high performance of multipeptide ELISAs provides a powerful argument for their use in trachoma control (24, 26, 27) and in screening for sexually transmitted infections (21, 25, 28). Such multipeptide ELISAs will increase the effectiveness of trachoma surveillance programs, and they will enable earlier identification and treatment of young women with chlamydial infection in sexually transmitted disease (STD) screening, reducing a woman’s likelihood of experiencing highly consequential reproductive health complications. In conclusion, specific and sensitive peptide ELISAs will improve *C. trachomatis* serology and will thereby help to more accurately diagnose and mitigate C. trachomatis infections and their sequelae (61–63, 66–70).

## MATERIALS AND METHODS

### Sera.

In this study, we used sera of 174 women that were collected from three cohorts: (i) 125 women with C. trachomatis infection confirmed by NAAT (53) whose sera were considered C. trachomatis positive; (ii) 18 healthy, low-risk women who had never been diagnosed with C. trachomatis infection (and whose sera were considered C. trachomatis negative); and (iii) 31 healthy women who were normal blood donors and self-reported to be free of infections (sera considered C. trachomatis negative) (BioIVT North America and Asia Pacific, Westbury, NY). Anti-Chlamydia trachomatis MIF titers (antigens A to I, K, and L1 to L3) were determined for 121 of the 125 C. trachomatis NAAT-positive women at the University of Washington. Serum samples of the healthy 18 women with low risk for C. trachomatis exposure tested negative for antibodies against C. trachomatis in an ELISA using C. trachomatis elementary bodies as antigen (54), and the 31 blood donor sera tested negative in four commercial anti-C. trachomatis IgG antibody ELISAs; these 49 sera were not tested by MIF. The ages of all study subjects ranged from 18 to 38 years, with an average of 22 years. Among the cohort of 125 C. trachomatis-positive women, 74 were African American; 28 Caucasian; and 23 of Hispanic, Asian, or mixed racial origin. The 18 low-risk women never diagnosed with C. trachomatis infection were all Caucasian. Of the blood donor cohort, 13 were African American; 10 Caucasian; and 8 of Hispanic, Asian, or mixed racial origin.

Written consent was obtained from all serum donors. Blood donor sera were collected at BioIVT facilities located in the United States, using BioIVT standard operating procedures approved by appropriate regulatory and ethics authorities. The study protocol for the remaining sera was approved by the institutional review boards for human research of the University of Pittsburgh and the University of North Carolina at Chapel Hill.

### Determination of anti-C. trachomatis IgG by using four commercial ELISAs.

The study sera were tested for anti-C. trachomatis IgG with the following four commercial ELISAs according to the manufacturers’ instructions: (i) GenWay Chlamydia trachomatis IgG ELISA (GenWay Biotech, Inc., San Diego, CA), using Chlamydia trachomatis elementary bodies as antigen; (ii) Serion Chlamydia trachomatis IgG ELISA (Serion Immunologics, Würzburg, Germany), using a proprietary recombinant C. trachomatis major outer membrane protein (MOMP) segment as antigen; (iii) Savyon Chlamydia trachomatis IgG ELISA (Savyon Diagnostics Ltd., Ashdod, Israel), using proprietary OmpA species-specific peptides of different *C. trachomatis* serovars; and (iv) Medac plus ELISA for Chlamydia trachomatis IgG (Medac GmbH, Wedel, Germany), using a proprietary C. trachomatis-specific MOMP variable domain peptide as antigen.

### C. trachomatis-specific peptide antigens.

Eleven C. trachomatis peptide antigens that had been identified previously (77) were used (Table 2). The amino acid sequences of these peptide antigens are highly conserved within the major clade strains of C. trachomatis (95% to 100% sequence identity [%SeqID]), except for OmpA and TarP peptides. Importantly, the sequences of these C. trachomatis peptides are highly evolutionarily divergent from those of the remaining 10 *Chlamydia* spp. and have only a marginal probability (≤0.01) of cross-reactivity with antibodies raised against non-C. trachomatis chlamydiae. Peptide antigens were chemically synthesized with N-terminal biotin followed by a serine-glycine-serine-glycine spacer and captured on streptavidin-coated white microtiter plates (Fisher Scientific, Roskilde, Denmark).

### Prediction of peptide cross-reactivity.

The probability of peptide cross-reactivity (*P*_cross_) was calculated from percent pairwise sequence divergence as follows: *P*_cross_ = *e*^(−9.4153 + 0.123223 × percent sequence identity)^/[1 + *e*^(−9.4153 + 0.123223 × percent sequence identity)^]. At <40%, 45%, 60%, 75%, and 90% sequence identity, this translates to *P*_cross_ values of 0.01, 0.02, 0.12, 0.46, and 0.84, respectively (Table 2). This probability of cross-reactivity of peptide antigens with antibodies against a heterologous B cell epitope had been described earlier based on a large experimental data set (43).

### Seroreactivity of C. trachomatis peptide antigens.

Primary antibodies were detected with horseradish peroxidase-conjugated secondary antibodies in chemiluminescent ELISA as described previously (77), with the following modifications: for wash buffer, 0.15 M NaCl, 20 mM Tris-HCl (pH 7.5), 0.025% Tween 20, and 0.001% benzalkonium chloride; for assay diluent, 0.125 M NaCl, 20 mM Tris-HCl (pH 7.5), 0.025% Tween 20, 2% rabbit serum, 0.2% bovine serum albumin, 0.2% casein, 0.2% polyethylene glycol, and 0.005% benzalkonium chloride; for blocking buffer, 0.125 M NaCl, 20 mM Tris-HCl (pH 7.5), 2% rabbit serum, 0.2% bovine serum albumin, 0.2% casein, 0.2% polyethylene glycol, and 0.005% benzalkonium chloride. The following monoclonal mouse anti-human antibody conjugates were purchased from Southern Biotech, Birmingham, AL: IgG1-HRP (catalog no. 9052-05), IgG3-HRP (catalog no. 9210-05), and IgA1-HRP (catalog no. B3506B4).

### IgG1, IgG3, and IgA1 reactivities of 11 C. trachomatis peptide antigens with 174 individual sera.

The set of 11 C. trachomatis peptide antigens (Table 2) was tested with a panel of individual sera of the 174 women described above. Monoclonal conjugates were used to detect human IgG1, IgG3, and IgA1 antibodies bound to C. trachomatis peptides. All tests were performed by using only a single peptide antigen with a single serum and a single conjugate per well.

### Composite reference standards (CRS) for C. trachomatis exposure and antibody status.

For evaluation of C. trachomatis exposure, a CRS was constructed by combining the results of NAAT and four commercial ELISA tests. A second CRS (consensus) for evaluation of all individual tests for determination of C. trachomatis antibody status was derived by defining any serum as antibody positive if the test result of any 1 of the following 7 tests was positive: GenWay, Serion, Savyon, and Medac ELISAs and the IgG1, IgG3, and IgA1 ELISAs combining the 11 C. trachomatis peptide antigens.

### Receiver operating characteristic (ROC) analysis.

ROC curves were plotted that displayed the true positive rate of an assay (sensitivity) as a function of the false-positive rate (1 − specificity). Fitted receiver operating characteristic (ROC) curves were constructed by use of freeware JavaScript programs JROCFIT and JLABROC4 (72). The ROC curves and the corresponding 95% confidence intervals (CI) were fitted by maximum likelihood estimation of a binormal model. The binormal model predicted the classification of the *X* variable, serum positivity/negativity, that was known *a priori* based on C. trachomatis infection status or anti-C. trachomatis antibody status. The observed continuous *Y* response variables were the values of serum reactivities expressed in relative light units (RLU) for 11 C. trachomatis peptides or optical density (OD) values for commercial ELISAs.

### Assay performance evaluation by area under the ROC curve (ROC-AUC).

The area under the ROC curve was used as the threshold-independent assay performance measure. An AUC value of 0.50 indicates that discrimination of positive from negative data equals random categorization (no discrimination), and an AUC value of 1.0 indicates that prediction is perfect (100% accurate discrimination). Following standard terminology for effect sizes (73), we defined the test discriminatory power for prediction of the C. trachomatis infection phenotype at AUCs of 0.90 to 1.00 as excellent, 0.80 to 0.90 as good, 0.70 to 0.80 as fair, 0.60 to 0.70 as poor, and 0.50 to 0.60 as failed.

### Determination of optimal peptide antigen combinations.

The best-performing and complementary sets of peptide antigens were identified also by ROC-AUC analyses. Based on observed IgG1, IgG3, and IgA1 seroreactivities (Log_2_ RLU) of the 11 individual C. trachomatis peptide antigens (predictor *Y* variable), discriminant analyses (JMP 11; SAS Institute, Cary, NC) predicted the anti-C. trachomatis antibody status *X* variable known *a priori*. In equal covariance matrices with linear fit models, the predictor *Y* variable with the maximum discriminatory power was entered stepwise into the ROC model for the *X* variable classification of 174 sera into the C. trachomatis-positive or -negative group. Thus, the most significant covariate among the as-yet-unentered individual peptide reactivities was included at each subsequent step. This approach resulted in determining the maximum predictive power of combinations of 2 through all 11 peptide antigens.

### Determination of likelihood ratios in diagnostic testing.

For evaluation of diagnostic anti-C. trachomatis antibody assay performance, positive-likelihood ratios (+LR) and negative-likelihood ratios (−LR) were calculated at 70% to 98% assay specificity cutoff values and the resultant assay sensitivity from ROC curves. The positive-likelihood ratio (+LR) is defined as the ratio between the probability of a positive test in subjects with the disease compared to those without the disease (74); i.e., +LR = true positive rate/false-positive rate. The negative-likelihood ratio (−LR) is defined as the ratio of the probability that a negative result would occur in subjects with the disease to the probability that the same result would occur in subjects without the disease (74); i.e., −LR = false-negative rate/true-negative rate. Based on accepted ranges of effect size (73–75), the performance of an assay was deemed diagnostically useful if both the +LR and −LR exceeded certain effect sizes as follows: large effect (+LR = ≥10, −LR = ≤0.10), moderate effect (+LR = ≥5, −LR = ≤0.15), and small effect (+LR = ≥2.5, −LR = ≤0.25).

### Determination of predictive value in dependence of anti-C. trachomatis antibody prevalence.

For population prevalence-dependent evaluation of assay performance, positive and negative predictive values (PPV and NPV) of individual assays were plotted against corresponding antibody prevalences. PPV and NPV for 0% to 100% prevalence were calculated from sensitivities and specificities in ROC curves as described earlier (73–75). The PPV was defined previously (75) as the probability that the disease is present when the test is positive as follows: PPV = [sensitivity × prevalence] ÷ [(sensitivity × prevalence) + (1 − specificity) × (1 − prevalence)]. The NPV was defined previously (75) as the probability that the disease is absent when the test is negative as follows: NPV = [specificity × (1 − prevalence)] ÷ [(1 − sensitivity) × prevalence + specificity × (1 − prevalence)]. The performance of an assay was considered high, moderate, or poor if both PPV and NPV exceeded 90.9%, 83.3%, or 71.4%, respectively. For direct quantitative comparisons of the diagnostic utility of the assays at 50% antibody prevalence, the point of equal sensitivities and specificities on the ROC curve was used to calculate PPV and NPV for each of the individual assays. For comparisons of the levels of diagnostic utility at low prevalence, a highly stringent cutoff (98% specificity) and the resultant sensitivity were uniformly chosen for all assays to calculate PPV and NPV.

### Statistical analyses.

Statistical analyses were performed and graphical outputs generated by use of either of the software packages Microsoft Excel 2016 (Microsoft, Redmond, WA) and Statistica 7.1 (StatSoft, Tulsa, OK). Antibody detection frequencies were compared by two-way Fisher's exact test. For comparisons of ROC-AUC values, corresponding sensitivities were calculated at specificity cutoff values of 98%, 95%, 90%, 85%, and 80%. For comparisons of likelihood ratios, corresponding −LR values were calculated for +LR values of 5, 10, 15, 20, and 25. For comparisons of predictive values within given anti-C. trachomatis antibody prevalence ranges, 5 PPVs and 5 NPVs of each assay were calculated, corresponding to 5 equidistant prevalences. Means were then compared by one-tailed paired Student’s *t* test.

## References

[1] Kuo CC, Stephens RS, Bavoil PM, Kaltenboeck B 2011 Genus I. *Chlamydia* Jones, rake and Stearns 1945, 55^AL^, vol 4, p 846–865. *In* Bergey’s manual of systematic bacteriology, 2nd ed. Springer, New York, NY.

[2] Leonard CA, Borel N 2014 Chronic chlamydial diseases: from atherosclerosis to urogenital infections. Curr Clin Microbiol Rep 1:61–72. doi:10.1007/s40588-014-0005-8.

[3] Pedersen LN, Herrmann B, Møller JK 2009 Typing *Chlamydia trachomatis*: from egg yolk to nanotechnology. FEMS Immunol Med Microbiol 55:120–130. doi:10.1111/j.1574-695X.2008.00526.x. 19281564

[4] Tuuminen T, Palomäki P, Paavonen J 2000 The use of serologic tests for the diagnosis of chlamydial infections. J Microbiol Methods 42:265–279. doi:10.1016/S0167-7012(00)00209-8. 11044570

[5] Broeze KA, Opmeer BC, Coppus SFPJ, Van Geloven N, Alves MFC, Ånestad G, Bhattacharya S, Allan J, Guerra-Infante MF, Den Hartog JE, Land JA, Idahl A, Van der Linden PJ, Mouton JW, Ng EH, Van der Steeg JW, Steures P, Svenstrup HF, Tiitinen A, Toye B, Van der Veen F, Mol BW 2011 *Chlamydia* antibody testing and diagnosing tubal pathology in subfertile women: an individual patient data meta-analysis. Hum Reprod Update 17:301–310. doi:10.1093/humupd/dmq060. 21227996

[6] Menon S, Stansfield SH, Walsh M, Hope E, Isaia L, Righarts AA, Niupulusu T, Temese SVA, Iosefa-Siitia L, Auvaa L, Tapelu SA, Motu MF, Suaalii-Sauni T, Timms P, Hill PC, Huston WM 2016 Sero-epidemiological assessment of *Chlamydia trachomatis* infection and sub-fertility in Samoan women. BMC Infect Dis 16:175. doi:10.1186/s12879-016-1508-0. 27102989 PMC4839085

[7] Puolakkainen M 2013 Laboratory diagnosis of persistent human chlamydial infection. Front Cell Infect Microbiol 3:99. doi:10.3389/fcimb.2013.00099. 24381934 PMC3865385

[8] Block S, Hedrick J, Hammerschlag MR, Cassell GH, Craft JC 1995 *Mycoplasma pneumoniae* and *Chlamydia pneumoniae* in pediatric community-acquired pneumonia: comparative efficacy and safety of clarithromycin vs. erythromycin ethylsuccinate. Pediatr Infect Dis J 14:471–477. doi:10.1097/00006454-199506000-00002. 7667050

[9] Kutlin A, Roblin PM, Hammerschlag MR 1998 Antibody response to *Chlamydia pneumoniae* infection in children with respiratory illness. J Infect Dis 177:720–724. doi:10.1086/514223. 9498453

[10] Wang SP, Grayston JT 1970 Immunologic relationship between genital TRIC, lymphogranuloma venereum, and related organisms in a new microtiter indirect immunofluorescence test. Am J Ophthalmol 70:367–374. doi:10.1016/0002-9394(70)90096-6. 4915925

[11] Wang SP, Kuo CC, Grayston JT 1973 A simplified method for immunological typing of trachoma-inclusion conjunctivitis-lymphogranuloma venereum organisms. Infect Immun 7:356–360. 16558075 10.1128/iai.7.3.356-360.1973 PMC422683

[12] Wang SP, Grayston JT 1974 Human serology in *Chlamydia trachomatis* infection with microimmunofluorescence. J Infect Dis 130:388–397. doi:10.1093/infdis/130.4.388. 4613759

[13] Campbell LA, Kuo CC, Grayston JT 1990 Structural and antigenic analysis of *Chlamydia pneumoniae*. Infect Immun 58:93–97. 2294060 10.1128/iai.58.1.93-97.1990 PMC258413

[14] Haralambieva I, Iankov I, Petrov D, Ivanova R, Kamarinchev B, Mitov I 2001 Cross-reaction between the genus-specific lipopolysaccharide antigen of *Chlamydia* spp. and the lipopolysaccharides of *Porphyromonas gingivalis*, *Escherichia coli* O119 and *Salmonella newington*: implications for diagnosis. Diagn Microbiol Infect Dis 41:99–106. doi:10.1016/S0732-8893(01)00299-1. 11750161

[15] Ozanne G, Lefebvre J 1992 Specificity of the microimmunofluorescence assay for the serodiagnosis of *Chlamydia pneumoniae* infections. Can J Microbiol 38:1185–1189. doi:10.1139/m92-194. 1477792

[16] Wong YK, Sueur JM, Fall CH, Orfila J, Ward ME 1999 The species specificity of the microimmunofluorescence antibody test and comparisons with a time resolved fluoroscopic immunoassay for measuring IgG antibodies against *Chlamydia pneumoniae*. J Clin Pathol 52:99–102. doi:10.1136/jcp.52.2.99. 10396235 PMC501051

[17] Wagenvoort JHT, Koumans D, Van de Cruijs M 1999 How useful is the *Chlamydia* micro-immunofluorescence (MIF) test for the gynaecologist? Eur J Obstet Gynecol Reprod Biol 84:13–15. doi:10.1016/S0301-2115(98)00303-0. 10413220

[18] Kern DG, Neill MA, Schachter J 1993 A seroepidemiologic study of *Chlamydia pneumoniae* in Rhode Island. Evidence of serologic cross-reactivity. Chest 104:208–213. 8325072 10.1378/chest.104.1.208

[19] Peeling RW, Wang SP, Grayston JT, Blasi F, Boman J, Clad A, Freidank H, Gaydos CA, Gnarpe J, Hagiwara T, Jones RB, Orfila J, Persson K, Puolakkainen M, Saikku P, Schachter J 2000 *Chlamydia pneumoniae* serology: interlaboratory variation in microimmunofluorescence assay results. J Infect Dis 181:S426–S429. doi:10.1086/315603. 10839729

[20] Rabenau HF, Köhler E, Peters M, Doerr HW, Weber B 2000 Low correlation of serology with detection of *Chlamydia trachomatis* by ligase chain reaction and antigen EIA. Infection 28:97–102. doi:10.1007/s150100050054. 10782395

[21] Bas S, Vischer TL 1998 *Chlamydia trachomatis* antibody detection and diagnosis of reactive arthritis. Br J Rheumatol 37:1054–1059. doi:10.1093/rheumatology/37.10.1054. 9825743

[22] Bas S, Muzzin P, Ninet B, Bornand JE, Scieux C, Vischer TL 2001 Chlamydial serology: comparative diagnostic value of immunoblotting, microimmunofluorescence test, and immunoassays using different recombinant proteins as antigens. J Clin Microbiol 39:1368–1377. doi:10.1128/JCM.39.4.1368-1377.2001. 11283058 PMC87941

[23] Bas S, Genevay S, Schenkel MC, Vischer TL 2002 Importance of species-specific antigens in the serodiagnosis of *Chlamydia trachomatis* reactive arthritis. Rheumatology 41:1017–1020. doi:10.1093/rheumatology/41.9.1017. 12209035

[24] West SK, Munoz B, Weaver J, Mrango Z, Dize L, Gaydos C, Quinn TC, Martin DL 2016 Can we use antibodies to *Chlamydia trachomatis* as a surveillance tool for national trachoma control programs? Results from a district survey. PLoS Negl Trop Dis 10:e0004352. doi:10.1371/journal.pntd.0004352. 26771906 PMC4714879

[25] Woodhall SC, Wills GS, Horner PJ, Craig R, Mindell JS, Murphy G, McClure MO, Soldan K, Nardone A, Johnson AM 2017 *Chlamydia trachomatis* Pgp3 antibody population seroprevalence before and during an era of widespread opportunistic *Chlamydia* screening in England (1994–2012). PLoS One 12:e0152810. doi:10.1371/journal.pone.0152810. 28129328 PMC5271337

[26] West SK, Munoz B, Kaur H, Dize L, Mkocha H, Gaydos CA, Quinn TC 2018 Longitudinal change in the serology of antibodies to *Chlamydia trachomatis* pgp3 in children residing in a trachoma area. Sci Rep 8:3520. doi:10.1038/s41598-018-21127-0. 29476106 PMC5824943

[27] Kaur H, Dize L, Munoz B, Gaydos C, West SK 2018 Evaluation of the reproducibility of a serological test for antibodies to *Chlamydia trachomatis* pgp3: a potential surveillance tool for trachoma programs. J Microbiol Methods 147:56–58. doi:10.1016/j.mimet.2018.02.017. 29501689

[28] Horner PJ, Wills GS, Righarts A, Vieira S, Kounali D, Samuel D, Winston A, Muir D, Dickson NP, McClure MO 2016 *Chlamydia trachomatis* Pgp3 antibody persists and correlates with self-reported infection and behavioural risks in a blinded cohort study. PLoS One 11:e0151497. doi:10.1371/journal.pone.0151497. 26974653 PMC4790965

[29] Myers GSA, Mathews SA, Eppinger M, Mitchell C, O’Brien KK, White OR, Benahmed F, Brunham RC, Read TD, Ravel J, Bavoil PM, Timms P 2009 Evidence that human *Chlamydia pneumoniae* was zoonotically acquired. J Bacteriol 191:7225–7233. doi:10.1128/JB.00746-09. 19749045 PMC2786552

[30] Donati M, Laroucau K, Storni E, Mazzeo C, Magnino S, Di Francesco A, Baldelli R, Ceglie L, Renzi M, Cevenini R 2009 Serological response to pgp3 protein in animal and human chlamydial infections. Vet Microbiol 135:181–185. doi:10.1016/j.vetmic.2008.09.037. 18945555

[31] Stothard DR, Williams JA, Van Der Pol B, Jones RB 1998 Identification of a *Chlamydia trachomatis* serovar E urogenital isolate which lacks the cryptic plasmid. Infect Immun 66:6010–6013. 9826386 10.1128/iai.66.12.6010-6013.1998 PMC108762

[32] Schachter J 2007 The *Chlamydia trachomatis* plasmid deletion mutant—what does it mean to us? Sex Transm Dis 34:257. doi:10.1097/OLQ.0b013e31805d0209. 17483724

[33] Närvänen A, Puolakkainen M, Hao W, Kino K, Suni J 1997 Detection of antibodies to *Chlamydia trachomatis* with peptide-based species-specific enzyme immunoassay. Infect Dis Obstet Gynecol 5:349–354. doi:10.1155/S1064744997000616. 18476184 PMC2364578

[34] Mygind P, Christiansen G, Persson K, Birkelund S 2000 Detection of *Chlamydia trachomatis*-specific antibodies in human sera by recombinant major outer membrane protein polyantigens. J Med Microbiol 49:457–465. doi:10.1099/0022-1317-49-5-457. 10798559

[35] Morré SA, Munk C, Persson K, Krüger-Kjaer S, van Dijk R, Meijer CJ, van den Brule AJ 2002 Comparison of three commercially available peptide-based immunoglobulin G (IgG) and IgA assays to microimmunofluorescence assay for detection of *Chlamydia trachomatis* antibodies. J Clin Microbiol 40:584–587. doi:10.1128/JCM.40.2.584-587.2002. 11825974 PMC153365

[36] Land JA, Gijsen AP, Kessels AGH, Slobbe MEP, Bruggeman CA 2003 Performance of five serological *Chlamydia* antibody tests in subfertile women. Hum Reprod 18:2621–2627. doi:10.1093/humrep/deg479. 14645182

[37] Komoda T 2007 Kinetic study of antibodies (IgG, IgA) to *Chlamydia trachomatis*: importance of IgA antibody in screening test for *C. trachomatis* infection by peptide-based enzyme immunosorbent assay. Jpn J Infect Dis 60:347–351. 18032832

[38] Verkooyen RP, Peeters MF, van Rijsoort-Vos JH, Van der Meijden WI, Mouton JW 2002 Sensitivity and specificity of three new commercially available *Chlamydia trachomatis* tests. Int J STD AIDS 13:23–25. doi:10.1258/095646202762226119. 12537721

[39] Frikha-Gargouri O, Gdoura R, Znazen A, Gargouri J, Rebai A, Hammami A 2009 Diagnostic value of an enzyme-linked immunosorbent assay using the recombinant CT694 species-specific protein of *Chlamydia trachomatis*. J Appl Microbiol 107:1875–1882. doi:10.1111/j.1365-2672.2009.04365.x. 19486214

[40] Wills GS, Horner PJ, Reynolds R, Johnson AM, Muir DA, Brown DW, Winston A, Broadbent AJ, Parker D, McClure MO 2009 Pgp3 antibody enzyme-linked immunosorbent assay, a sensitive and specific assay for seroepidemiological analysis of *Chlamydia trachomatis* infection. Clin Vaccine Immunol 16:835–843. doi:10.1128/CVI.00021-09. 19357314 PMC2691054

[41] Stansfield SH, Patel P, Debattista J, Armitage CW, Cunningham K, Timms P, Allan J, Mittal A, Huston WM 2013 Proof of concept: a bioinformatic and serological screening method for identifying new peptide antigens for *Chlamydia trachomatis* related sequelae in women. Results Immunol 3:33–39. doi:10.1016/j.rinim.2013.05.001. 24600556 PMC3908335

[42] Menon S, Stansfield SH, Logan B, Hocking JS, Timms P, Rombauts L, Allan JA, Huston WM 2016 Development and evaluation of a multi-antigen peptide ELISA for the diagnosis of *Chlamydia trachomatis*-related infertility in women. J Med Microbiol 65:915–922. doi:10.1099/jmm.0.000311. 27430220

[43] Rahman KS, Chowdhury EU, Poudel A, Ruettger A, Sachse K, Kaltenboeck B 2015 Defining species-specific immunodominant B cell epitopes for molecular serology of *Chlamydia* species. Clin Vaccine Immunol 22:539–552. doi:10.1128/CVI.00102-15. 25761461 PMC4412952

[44] Li Z, Chen C, Chen D, Wu Y, Zhong Y, Zhong G 2008 Characterization of fifty putative inclusion membrane proteins encoded in the *Chlamydia trachomatis* genome. Infect Immun 76:2746–2757. doi:10.1128/IAI.00010-08. 18391011 PMC2423075

[45] Wang J, Zhang Y, Lu C, Lei L, Yu P, Zhong G 2010 A genome-wide profiling of the humoral immune response to *Chlamydia trachomatis* infection reveals vaccine candidate antigens expressed in humans. J Immunol 185:1670–1680. doi:10.4049/jimmunol.1001240. 20581152

[46] Rahman KhS, Chowdhury EU, Sachse K, Kaltenboeck B 2016 Inadequate reference data sets biased towards short non-epitopes confound B-cell epitope prediction. J Biol Chem 291:14585–14599. doi:10.1074/jbc.M116.729020. 27189949 PMC4938180

[47] Sachse K, Rahman KS, Schnee C, Müller E, Peisker M, Schumacher T, Schubert E, Ruettger A, Kaltenboeck B, Ehricht R 2018 A novel synthetic peptide microarray assay detects *Chlamydia* species-specific antibodies in animal and human sera. Sci Rep 8:4701. doi:10.1038/s41598-018-23118-7. 29549361 PMC5856796

[48] Brack C, Hirama M, Lenhard-Schuller RL, Tonegawa S 1978 A complete immunoglobulin gene is created by somatic recombination. Cell 15:1–14. doi:10.1016/0092-8674(78)90078-8. 100225

[49] Hadgu A, Miller W 2001 Using a combination of reference tests to assess the accuracy of a diagnostic test by A Alonzo and M Pepe, Statistics in Medicine 1999; 18: 2987-3003. Stat Med 20:656–660. doi:10.1002/sim.762. 11223907

[50] Baughman AL, Bisgard KM, Cortese MM, Thompson WW, Sanden GN, Strebel PM 2008 Utility of composite reference standards and latent class analysis in evaluating the clinical accuracy of diagnostic tests for pertussis. Clin Vaccine Immunol 15:106–114. doi:10.1128/CVI.00223-07. 17989336 PMC2223853

[51] Schiller I, van Smeden M, Hadgu A, Libman M, Reitsma JB, Dendukuri N 2016 Bias due to composite reference standards in diagnostic accuracy studies. Stat Med 35:1454–1470. doi:10.1002/sim.6803. 26555849

[52] Naaktgeboren CA, van der Zwaan GJ 2015 Improving the methodological framework for diagnostic studies. PhD thesis Utrecht University, Utrecht, Netherlands.

[53] Russell AN, Zheng X, O’Connell CM, Wiesenfeld HC, Hillier SL, Taylor BD, Picard MD, Flechtner JB, Zhong W, Frazer LC, Darville T 2016 Identification of *Chlamydia trachomatis* antigens recognized by T cells from highly exposed women who limit or resist genital tract infection. J Infect Dis 214:1884–1892. doi:10.1093/infdis/jiw485. 27738051 PMC5142095

[54] Albritton HL, Kozlowski PA, Lillis RA, McGowin CL, Siren JD, Taylor SN, Ibana JA, Buckner LR, Shen L, Quayle AJ 2017 A novel whole-bacterial enzyme linked-immunosorbant assay to quantify *Chlamydia trachomatis* specific antibodies reveals distinct differences between systemic and genital compartments. PLoS One 12:e0183101. doi:10.1371/journal.pone.0183101. 28797112 PMC5552291

[55] Boelaert M, Aoun K, Liinev J, Goetghebeur E, Van Der Stuyft P 1999 The potential of latent class analysis in diagnostic test validation for canine *Leishmania infantum* infection. Epidemiol Infect 123:499–506. doi:10.1017/S0950268899003040. 10694163 PMC2810786

[56] Steiner AZ, Diamond MP, Legro RS, Schlaff WD, Barnhart KT, Casson PR, Christman GM, Alvero R, Hansen KR, Geisler WM, Thomas T, Santoro N, Zhang H, Eisenberg E; Reproductive Medicine Network 2015 *Chlamydia trachomatis* immunoglobulin G3 seropositivity is a predictor of reproductive outcomes in infertile women with patent fallopian tubes. Fertil Steril 104:1522–1526. doi:10.1016/j.fertnstert.2015.08.022. 26413816 PMC4663111

[57] Collins AM, Jackson KJ 2013 A temporal model of human IgE and IgG antibody function. Front Immunol 4:235. doi:10.3389/fimmu.2013.00235. 23950757 PMC3738878

[58] Bas S, Muzzin P, Vischer TL 2001 *Chlamydia trachomatis* serology: diagnostic value of outer membrane protein 2 compared with that of other antigens. J Clin Microbiol 39:4082–4085. doi:10.1128/JCM.39.11.4082-4085.2001. 11682533 PMC88490

[59] Goodhew EB, Priest JW, Moss DM, Zhong G, Munoz B, Mkocha H, Martin DL, West SK, Gaydos C, Lammie PJ 2012 CT694 and pgp3 as serological tools for monitoring trachoma programs. PLoS Negl Trop Dis 6:e1873. doi:10.1371/journal.pntd.0001873. 23133684 PMC3486877

[60] Gwyn S, Mitchell A, Dean D, Mkocha H, Handali S, Martin DL 2016 Lateral flow-based antibody testing for *Chlamydia trachomatis*. J Immunol Methods 435:27–31. doi:10.1016/j.jim.2016.05.008. 27208400

[61] Skwor T, Kandel RP, Basravi S, Khan A, Sharma B, Dean D 2010 Characterization of humoral immune responses to chlamydial HSP60, CPAF, and CT795 in inflammatory and severe trachoma. Invest Ophthalmol Vis Sci 51:5128–5136. doi:10.1167/iovs.09-5113. 20463311 PMC3066612

[62] Rantsi T, Joki-Korpela P, Wikström E, Öhman H, Bloigu A, Lehtinen M, Gissler M, Tiitinen A, Paavonen J, Surcel HM 2016 Population-based study of prediagnostic antibodies to *Chlamydia trachomatis* in relation to adverse pregnancy outcome. Sex Transm Dis 43:382–387. doi:10.1097/OLQ.0000000000000432. 27196260

[63] Rodgers AK, Budrys NM, Gong S, Wang J, Holden A, Schenken RS, Zhong G 2011 Genome-wide identification of *Chlamydia trachomatis* antigens associated with tubal factor infertility. Fertil Steril 96:715–721. doi:10.1016/j.fertnstert.2011.06.021. 21742324 PMC3225487

[64] Andresen H, Grötzinger C, Zarse K, Kreuzer OJ, Ehrentreich-Förster E, Bier FF 2006 Functional peptide microarrays for specific and sensitive antibody diagnostics. Proteomics 6:1376–1384. doi:10.1002/pmic.200500343. 16456884 PMC7167710

[65] Ehricht R, Adelhelm K, Monecke S, Huelseweh B 2009 Application of protein arraytubes to bacteria, toxin, and biological warfare agent detection. Methods Mol Biol 509:85–105. doi:10.1007/978-1-59745-372-1_6. 19212716

[66] Budrys NM, Gong S, Rodgers AK, Wang J, Louden C, Shain R, Schenken RS, Zhong G 2012 *Chlamydia trachomatis* antigens recognized in women with tubal factor infertility, normal fertility, and acute infection. Obstet Gynecol 119:1009–1016. doi:10.1097/AOG.0b013e3182519326. 22525912 PMC4608258

[67] Hjelholt A, Christiansen G, Johannesson TG, Ingerslev HJ, Birkelund S 2011 Tubal factor infertility is associated with antibodies against *Chlamydia trachomatis* heat shock protein 60 (HSP60) but not human HSP60. Hum Reprod 26:2069–2076. doi:10.1093/humrep/der167. 21642639

[68] Hokynar K, Korhonen S, Norja P, Paavonen J, Puolakkainen M 2017 Antibody to *Chlamydia trachomatis* proteins, TroA and HtrA, as a biomarker for *Chlamydia trachomatis* infection. Eur J Clin Microbiol Infect Dis 36:49–56. doi:10.1007/s10096-016-2769-7. 27638011

[69] Lu C, Holland MJ, Gong S, Peng B, Bailey RL, Mabey DW, Wu Y, Zhong G 2012 Genome-wide identification of *Chlamydia trachomatis* antigens associated with trachomatous trichiasis. Invest Ophthalmol Vis Sci 53:2551–2559. doi:10.1167/iovs.11-9212. 22427578 PMC3366722

[70] Pickering H, Burr SE, Derrick T, Makalo P, Joof H, Hayward RD, Holland MJ 2017 Profiling and validation of individual and patterns of *Chlamydia trachomatis*-specific antibody responses in trachomatous trichiasis. Parasit Vectors 10:143. doi:10.1186/s13071-017-2078-8. 28288672 PMC5347170

[71] Solomon AW 2006 World Health Organization and International Trachoma Initiative. Trachoma control: a guide for programme managers. http://apps.who.int/iris/bitstream/handle/10665/43405/9241546905_eng.pdf;jsessionid=E324284267101B9F7B60C38EFA558E7F?sequence=1.

[72] Eng J 2014 ROC analysis: Web-based calculator for ROC curves. Johns Hopkins University, Baltimore, MD Updated 19 3 2014 Accessed 4 January 2017 http://www.jrocfit.org.

[73] Šimundić AM 2009 Measures of diagnostic accuracy: basic definitions. EJIFCC 19:203–211. 27683318 PMC4975285

[74] Okeh UM, Okoro CN 2012 Evaluating measures of indicators of diagnostic test performance: fundamental meanings and formulators. J Biom Biostat 3:132 https://www.omicsonline.org/evaluating-measures-of-indicators-of-diagnostic-test-performance-fundamental-meanings-and-formulars-2155-6180.1000132.php?aid=4054.

[75] Maxim LD, Niebo R, Utell MJ 2014 Screening tests: a review with examples. Inhal Toxicol 26:811–828. doi:10.3109/08958378.2014.955932. 25264934 PMC4389712

[76] Hammerschlag MR 2003 Pneumonia due to *Chlamydia pneumoniae* in children: epidemiology, diagnosis, and treatment. Pediatr Pulmonol 36:384–390. doi:10.1002/ppul.10326. 14520720

[77] Rahman KS, Darville T, Russell AN, O’Connell CM, Wiesenfeld HC, Hillier SL, Chowdhury EU, Juan Y-C, Kaltenboeck B 2018 Discovery of human-specific immunodominant *Chlamydia trachomatis* B cell epitopes. mSphere 3:e00246-18. doi:10.1128/mSphere.00246-18. 30068558 PMC6070735

