# Supplementary material for: Comprehensive Molecular Serology of Human Chlamydia trachomatis Infections by Peptide Enzyme-Linked Immunosorbent Assays
Source: mSphere. 2018 Aug 1;3(4):e00253-18. doi: 10.1128/mSphere.00253-18 (PMC6070734; doi:10.1128/mSphere.00253-18)
Supplement: FIG S1 [file sph004182605sf1.pdf]

# SUPPLEMENTAL DATA

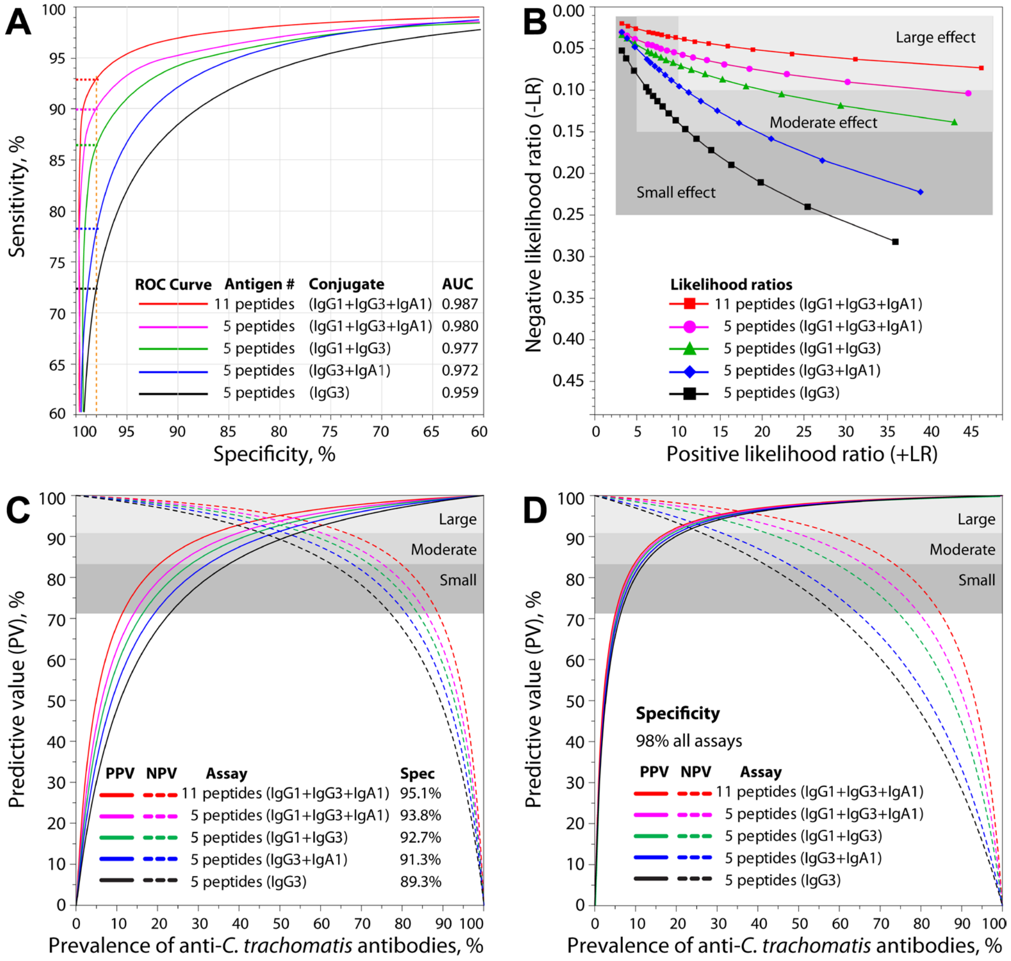


**FIG S1** Utility of *C. trachomatis* peptide antigen assays evaluated by ROC curves, likelihood ratios and predictive values. Performance differences were determined between *C. trachomatis* peptide antigen assays using the complete set of 11 peptides and the IgG1+IgG3+IgA1 conjugate combination versus assays with the reduced set of the 5 most informative peptides and different conjugate combinations. The antibody consensus of 125 *C. trachomatis* infection-positive and 49 -negative sera was used as categorical variable known *a priori*, and the observed values of serum reactivities of a test were used as predictor variables of the anti-*C. trachomatis* antibody status. The average $Log_2$ RLU signals of the peptide antigens for different combinations of conjugates were used as predictor variables. For reference, the green lines indicating the 5-peptide assay with IgG1+IgG3 detection correspond to those in Fig. 3 and Fig. 4. (A) ROC evaluation of individual anti-*C. trachomatis* antibody assays against the gold standard consensus antibody status. Solid color lines indicate maximum likelihood-fitted ROC curves. The dotted lines indicate sensitivity at 98% specificity. (B) Positive and negative likelihood ratios are independent of population prevalence of anti-*C. trachomatis* antibodies. Sensitivities were calculated at specificities ranging from 70% (left) to 90% (right). Using sensitivity and specificity data, positive (+LR) and negative likelihood ratios (-LR) were calculated. (C) and (D) Positive and negative predictive values in dependence of anti-*C. trachomatis* antibody population prevalence. (C) PPV, NPV, sensitivity, and specificity are equal for each assay at assumed high 50% antibody prevalence, but higher for the IgG1+IgG3+IgA1 reactivities of 11 peptide antigens (PPV = NPV = sensitivity = specificity = 95.1%) than for the conjugate combinations with 5 peptides. (D) In modeling for lower antibody prevalence, 98% specificity was chosen for all assays. The assay with 11 peptides and 3 conjugates also showed the highest performance.
